# Supplementary material for: Air-Frying Is a Better Thermal Processing Choice for Improving Antioxidant Properties of Brassica Vegetables
Source: Antioxidants (Basel). 2023 Feb 15;12(2):490. doi: 10.3390/antiox12020490 (PMC9952021; doi:10.3390/antiox12020490)
Supplement: Supplementary file 1 [file antioxidants-12-00490-s001.zip › antioxidants-2220017-supplementary.pdf]

Supplementary Data

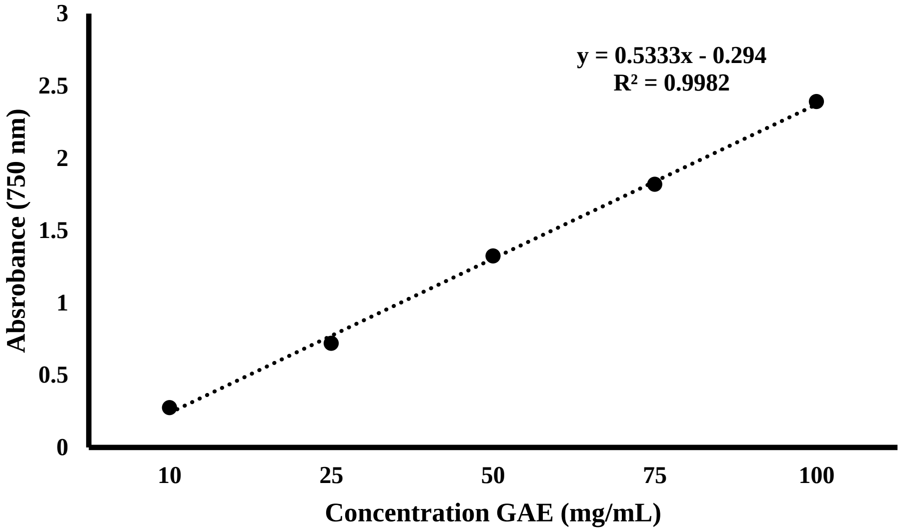

Figure S1: Total phenolic content standard curve using gallic acid solution (1 mM) as standard (GAE—gallic acid equivalents, mg—miligram, mL—mililiter, nm—nanometer, R^2—coefficient of variance)

Supplementary Data

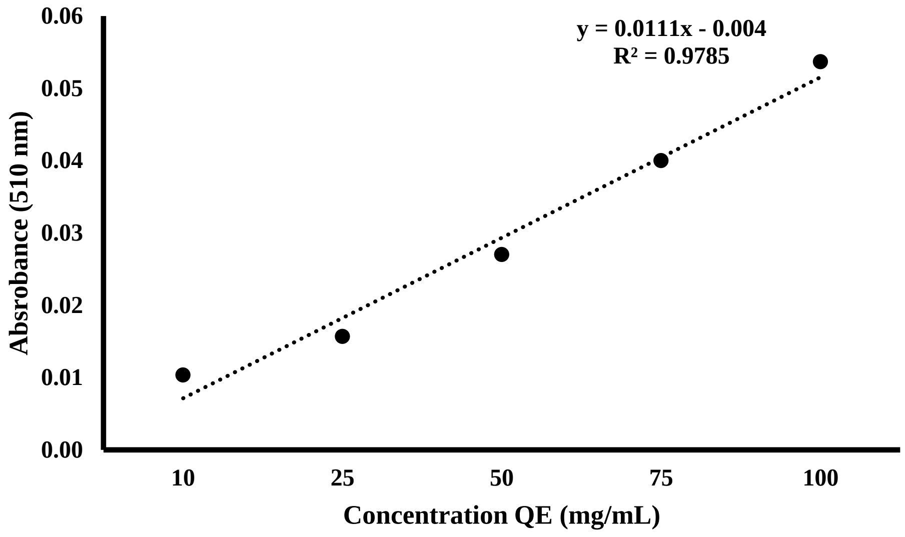

Figure S2: Total flavonoid content standard curve using quercetin solution (1 mM) as standard (QE—qercetin equivalents, nm—nanometer, mg—miligram, mL—mililiter, R^2—coefficient of variance)

Supplementary Data

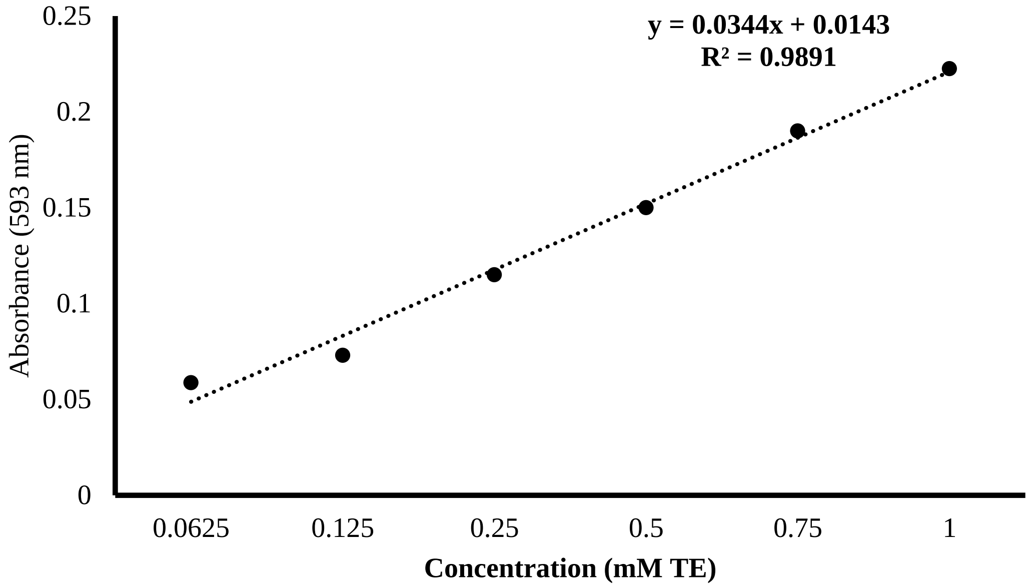

Figure S3: Standard curve for antioxidant activity by FRAP using trolox stnadard solution (1 mM) (TE—trolox equivalents, nm—nanometer, mg—miligram, mL—mililiter, R^2—coefficient of variance)

Supplementary Data

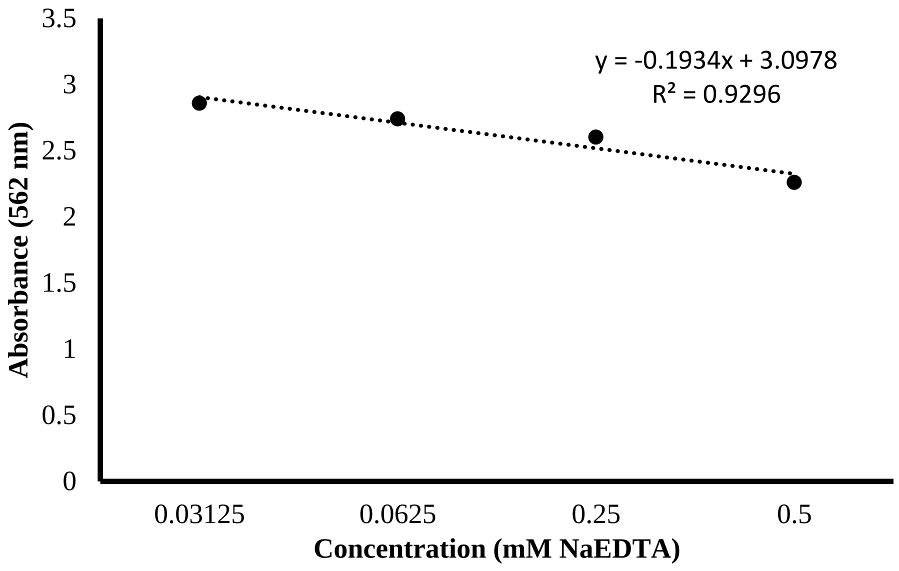

Figure S4: Standard curve for antioxidant activity by metal ion chelating activity using Na_2EDTA stnadard solution (EDTAE—EDTA equivalents, nm—nanometer, mg—miligram, mL—mililiter, R^2—coefficient of variance)

Supplementary Data

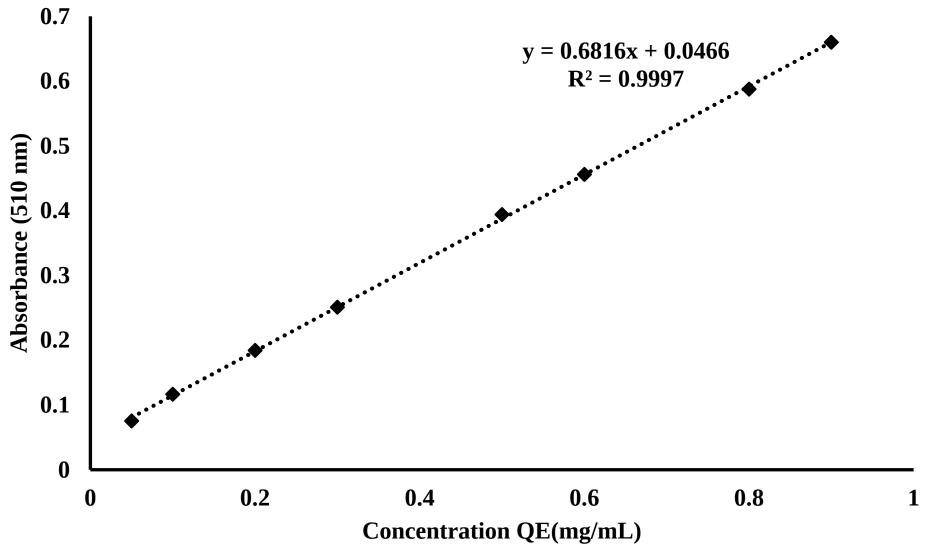

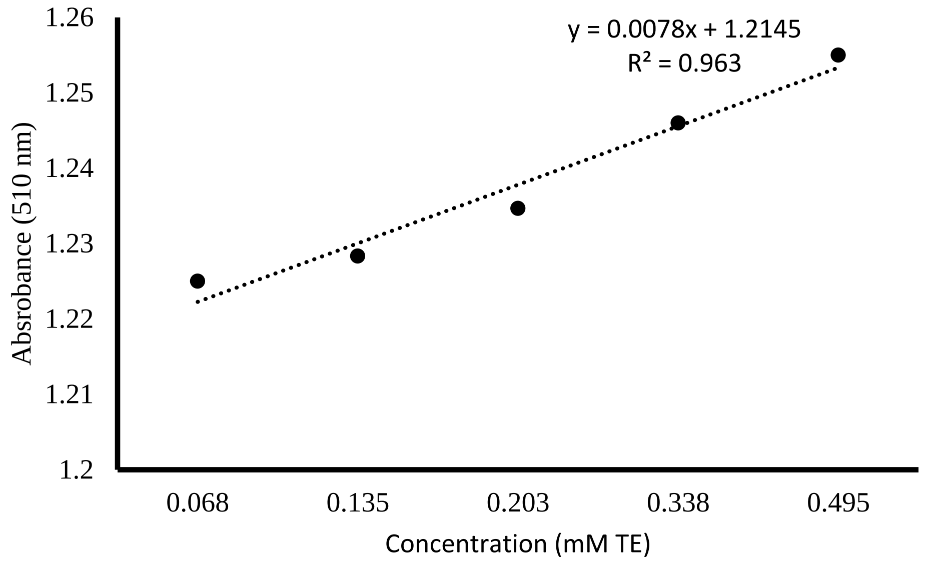

Figure S5: Standard curve for antioxidant activity by ABTS activity using trolox stnadard solution (TE—trolox equivalents, nm—nanometer, mg—miligram, mL—mililiter, R^2—coefficient of variance)
